# Preparation of Highly Antibacterial Polyethersulfone/Sulfonated Polyethersulfone Blend Composite Membrane and Research on Its Dye Separation Performance

**DOI:** 10.3390/molecules30040781

**Published:** 2025-02-08

**Authors:** Na Meng, Jialing Mi, Xuan Chen, Jinxin Liu, Hualei Zhu, Xiaoyu Zheng

**Affiliations:** 1 Jiangsu Key Laboratory of Industrial Pollution Control and Resource Reuse, School of Environment Engineering, Xuzhou University of Technology, Xuzhou 221018, China; 17751282269@163.com (J.M.); ljx2667495913@163.com (J.L.); 15298379691@163.com (H.Z.); 2 School of Technology-Saint Pertersburg Joint Engineering, Xuzhou University of Technology, Xuzhou 221018, China; chenxuan2645@163.com (X.C.); 19711126026@163.com (X.Z.)

**Keywords:** PES, SPES, composite membrane, dye separation, antibacterial properties

## Abstract

Bringing sulfonic acid groups into conventional polyethersulfone (PES) materials to prepare PES/sulfonated polyethersulfone (SPES) composite membranes has been shown to markedly enhance the hydrophilicity of the membranes and boost their separation efficiency in water treatment applications. However, membrane fouling due to microbial activity remains a critical challenge in the practical use of these membranes. Despite this, research into the antibacterial capabilities of PES/SPES composite membranes is scarce. This study employed the nonsolvent-induced phase inversion technique to prepare PES/SPES blend membranes and examined how the solid content influences their microstructure and separation performance. The findings indicated that an increase in solid content leads to higher casting solution viscosity, a reduction in membrane porosity from 10.8% to 4.06%, an increase in tensile strength from 0.79 MPa to 5.06 MPa, and a significant improvement in methylene blue rejection from 50% to nearly 100%. Additionally, the study revealed that the incorporation of SPES into the blend membranes enhances their resistance to bacterial adhesion, effectively suppressing microbial growth. Notably, the higher the sulfonic acid group content, the better the antibacterial properties of the composite membranes.

## 1. Introduction

As the dye industry continues to expand, the annual discharge of dye-containing wastewater is on the rise, exacerbating the pollution and destruction of the ecological environment. Consequently, the development of highly efficient and straightforward technologies for treating dye wastewater is crucial for safeguarding the environment and ensuring sustainable development [1,2]. Membrane separation technology stands out as an advanced wastewater treatment method that capitalizes on the selective permeability of membranes to effectively eliminate pollutants from water [3,4,5]. This approach not only achieves remarkable separation outcomes but also enables the recycling and reuse of the filtrate, thereby showcasing significant potential and advantages in dye wastewater management. Given its ease of operation and high efficiency, membrane separation technology has been extensively implemented in the recovery of dyes and the purification of wastewater. It has emerged as one of the pivotal technologies propelling the green transformation of the dye industry [6].

PES, renowned for its exceptional chemical stability and mechanical properties, holds a significant position in the realm of membrane materials [7,8,9,10]. However, its limited hydrophilicity has been a constraint in certain water treatment applications [11]. To improve this, researchers have prepared SPES, a modification that introduces sulfonic acid groups (-SO_3_H) to the PES molecular structure, thereby enhancing its hydrophilic properties [12,13,14]. Liu et al. utilized PES, sulfonated polysulfone (SPSf), and sulfonated SPES to prepare PES/SPSf/SPES blend loose nanofiltration membranes via the non-solvent induced phase separation (NIPS) technique. They explored the influence of SPES content on the structure of the membrane and its performance in dye/salt separation. Their findings revealed that with a 10% SPES content, the resulting nanofiltration membranes achieved a pure water permeability of 605 L/(m^2^·h·MPa) and retention rates for Disperse Red 74 (DR74) and Disperse Blue 79 (DB79) exceeding 99% [15]. Zhao et al. selected an SPES solution with a 14% sulfonation degree to prepare PES-SPES blend membranes and investigated the impact of the SPES to PES mass ratio on the desalination performance and antifouling characteristics of the blend membranes. They discovered that at an SPES to PES mass ratio of 0.75, the blend membranes exhibited a water flux of 253.7 L/m^2^·h and a retention rate for PEG20000 of 90.6% [16]. Klaysom et al. [5] prepared sulfonated SPES proton exchange membranes, which, upon investigation, were found to not only improve hydrophilicity but also to possess enhanced proton conductivity and selectivity [17]. The incorporation of sulfonic acid groups has significantly boosted the hydrophilicity and separation efficiency of the membrane in water treatment processes. However, membrane fouling due to microbial activity remains a critical challenge, as it diminishes the separation efficiency, shortens the lifespan of the membrane, and escalates operational costs. Despite this, research into the antibacterial capabilities of PES/SPES composite membranes remains scarce.

This paper employs PES and SPES as the primary materials and water as the coagulation medium to prepare PES/SPES blend composite membranes via the nonsolvent-induced phase inversion technique. It delves into the effects of varying solid content on the microstructure and dye retention capabilities of these composite membranes. Furthermore, it investigates the influence of -SO_3_H content on the antibacterial properties of the membranes. The goal is to prepare a PES/SPES blend composite membrane that not only excels in dye separation performance but also possesses robust antibacterial characteristics. This advancement is expected to enhance the resistance of the membrane to fouling and extend its lifespan, offering novel strategies for the development of superior SPES antibacterial membranes.

## 2. Results and Discussion

### 2.1. Calculation of Blend Membrane Compatibility

Blend compatibility, which is the capacity of various substances to integrate and form a homogeneous and stable system [18], is a critical assessment criterion for the construction of polymer blend systems. It is also a vital precondition for the preparation and structural regulation of polymeric porous membranes [19]. According to the Flory-Huggins theory, the enthalpy of mixing serves as a quantifiable measure of blend compatibility, with the formula as follows:

(1) ΔHm=X1M1ρ1(δ1−δ2)2X2X1M2ρ2+X2M1ρ112

where $\Delta H_{m}$ is the enthalpy of mixing; *X*_1_ and *X*_2_ denote mass fractions of polymers PES and SPES, respectively, and *X*_1_ + *X*_2_ = 1; M is the unit molar mass, g/mol; ρ is density, g/cm^2^; and δ is the solubility parameter, J^1/2^/mol^1/2^.

According to Schneier’s theory, when $\Delta H_{m}$ < 0.01 cal·mol^−1^, the blend system is deemed to be completely compatible. On the contrary, it is not completely compatible. Relevant parameters of the polymers in the PES/SPES system are shown in Table 1.

**Figure 1:**
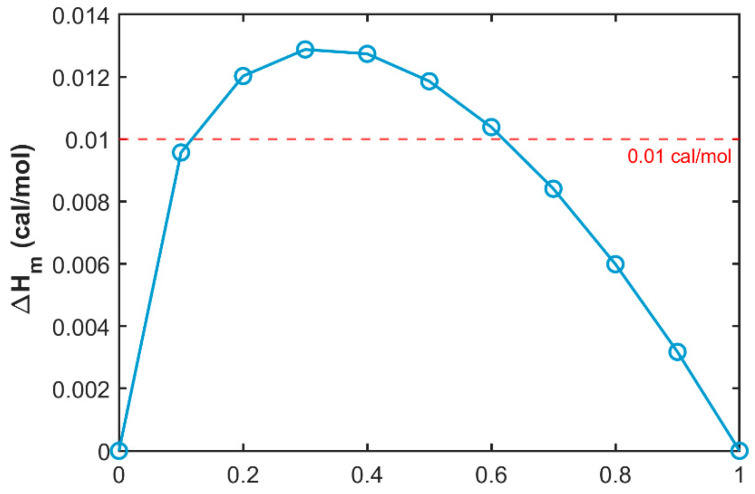
Enthalpy of mixing of PES in PES/SPES blend system.

Using Formula (9), the enthalpy of mixing for various blend ratios within the system was calculated, with the results depicted in Figure 1. It is observed that for the PES/SPES blend system, compatibility is relatively good when the mass fraction of PES is below approximately 10% or above 60%. The dashed line indicates the critical boundary between compatibility and incompatibility based on the Schiebel theory, with the region above the line representing the incompatible zone and the region below it representing the compatible zone. When the PES/SPES blend ratio is less than approximately 0.1 or greater than 0.6, the blend membrane system exhibits better compatibility. Consequently, this study selected a 60% PES composition as the fundamental condition for membrane preparation.

### 2.2. Chemical Structure Analysis of PES/SPES Blend Membrane

**Figure 2:**
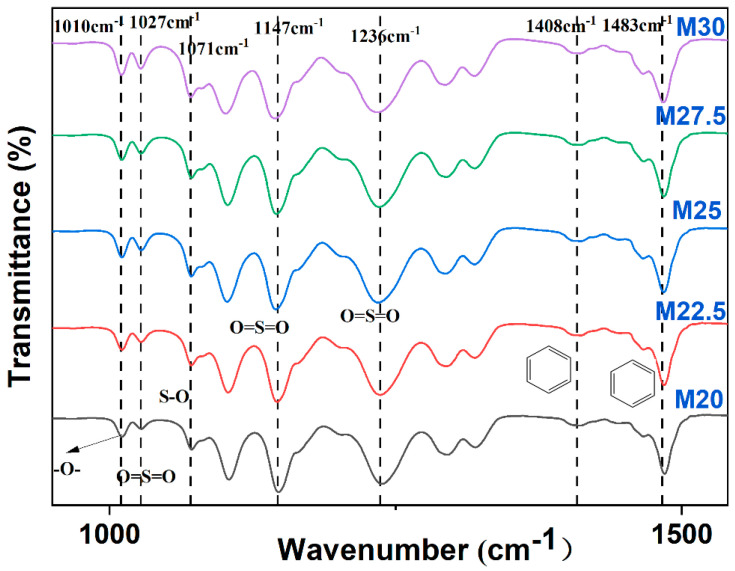
FTIR diagram of PES/SPES blend membrane (M20–M30).

**Figure 3:**
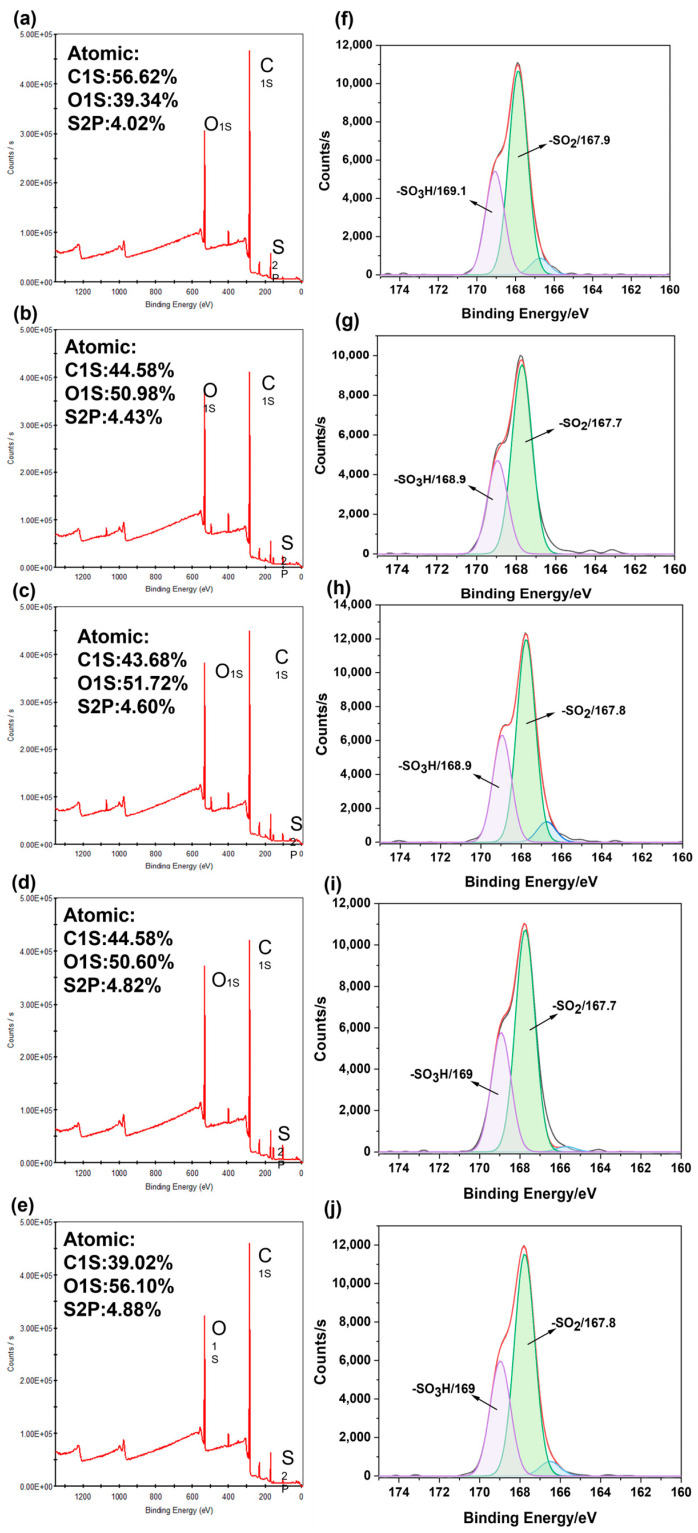
XPS survey (**a**–**e**) and analysis of XPS elements in the S2p region (**f**–**j**) of PES/SPES blend membrane.

The infrared spectroscopy results for the PES/SPES blend membrane are shown in Figure 2. The peak at 1010 cm⁻^1^ corresponds to the symmetric vibration absorption peak of the -O- group. Significant characteristic peaks are observed at 1027 cm⁻^1^, 1147 cm⁻^1^, and 1236 cm⁻^1^, which correspond to the symmetric and asymmetric vibration absorption peaks of the O=S=O group, respectively. The peaks at 1408 cm⁻^1^ and 1483 cm⁻^1^ correspond to the skeletal vibration absorption peaks of the benzene ring. Notably, the appearance of the S-O vibration absorption peak at 1071 cm⁻^1^ and the hydroxyl stretching vibration peak near 3400 cm⁻^1^ confirm the presence of the -SO_3_H group [16]. The details are as follows:

Figure 3f–j reveal the presence of sulfonic acid groups (-SO_3_H) at 169 eV on the surface of the blend membranes, confirming the existence of (-SO_3_H) on the membrane surface. Figure 3a–e present the full spectra of the blend membranes with varying concentrations, showing that the surfaces are predominantly composed of C, O, and S elements. It is observed that the content of carbon (C) decreases with increasing concentrations of SPES, while the content of oxygen (O) and sulfur (S) elements increases. The enrichment of (-SO_3_H) groups on the blend membrane surface leads to a relative reduction in C content. The full spectra also indicate a gradual increase in sulfur content, suggesting that during the phase separation process, the hydrophilic sulfonic acid groups from the casting solution migrate and accumulate on the blend membrane surface, with significant changes in the content of S and C elements. The migration and aggregation of sulfonic acid groups on the surface also promote the extension of sulfone groups from the SPES main chain onto the membrane surface. Consequently, the XPS analysis of the chemical composition on the blend membrane surface demonstrates that the preparation method for the new PES/SPES blend membrane successfully enriches hydrophilic sulfonic acid groups on the membrane surface, which is beneficial for enhancing the hydrophilicity of the blend membrane. 

### 2.3. Acidity and Viscosity Analysis of PES/SPES Blend Membrane

**Figure 4:**
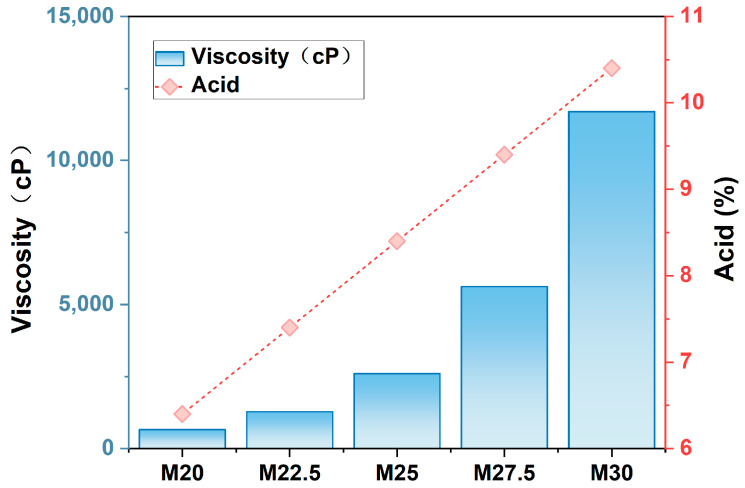
Change chart of viscosity and acidity of PES/SPES blend membrane.

Figure 4 illustrates the acidity and viscosity of SPES/SPES membranes with varying solid contents. As observed in Figure 4, viscosity increases in direct proportion to the solid content, rising gradually with each increment in solid content. At a solid content of 20%, the solution exhibits relatively low viscosity, whereas at 30%, the viscosity soars to approximately 12,000 cP. Concurrently, an increase in the solid content within the casting solution is also associated with heightened acidity, a result of the increased degree of sulfonation [16].

### 2.4. Morphological Characterization of PES/SPES Blend Membrane

**Figure 5:**
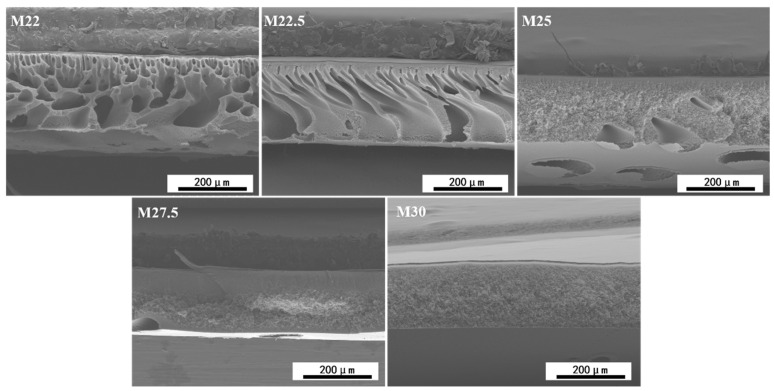
Diagram of cross-section SEM characterization of blend membrane.

Utilizing SEM, the cross-sectional microstructures of the prepared PES/SPES blend membranes were meticulously examined, as depicted in Figure 5. The blend membranes are composed of a dense skin layer and a porous support layer. With an increase in solid content, the structure of the support layer evolves from finger-like pores to a sponge-like morphology. At polymer contents of 22% and 22.5%, the viscosity of the casting solution is comparatively low, and the hydrophilicity of SPES predominantly influences phase separation, leading to a rapid phase separation rate and the formation of finger-like pore structures in the blend membranes. As the polymer content incrementally rises from 22.5% to 30.0%, the viscosity of the casting solution increases significantly due to the acid introduced by SPES [21], which becomes the predominant factor in phase separation, slowing the rate and resulting in a sponge-like structure for the M25, M27.5, and M30 membranes. Consequently, as the solid content in the casting solution increases, its viscosity also increases, causing the phase separation process to shift from being primarily controlled by hydrophilicity to being governed by the viscosity of the casting solution, and the support layer structure of the blend membranes transitions progressively from finger-like pores to a sponge-like configuration.

### 2.5. Characterization of Physical Properties of PES/SPES Blend Membrane

**Figure 6:**
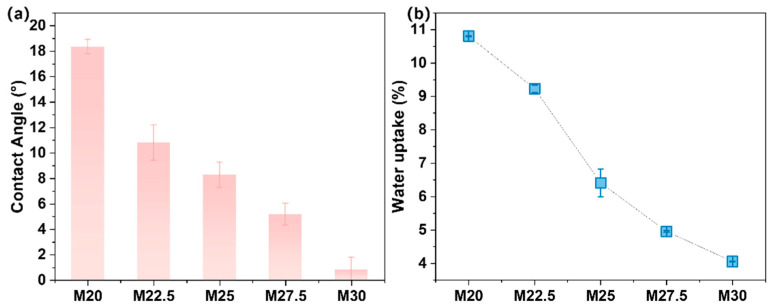
Changes in contact angle (**a**) and water absorption rate (**b**) of PES/SPES blend membrane with total solid content.

Figure 6a illustrates that the contact angle of the PES/SPES blend membrane progressively decreases with the elevation of solid content, signifying increased hydrophilicity. At 30% concentration, the contact angle is effectively negligible, nearly approaching 0°. The introduction of sulfonic acid groups, which are strongly hydrophilic, enhances the hydrophilicity of the blend membrane, facilitating a reduction in the contact angle and consequently improving the wettability and separation efficiency of the membrane. The water absorption rate is indicative of the porosity of the membrane; as depicted in Figure 6b, the water content of the blend membrane decreases from 10.8% to 4.06% with an increase in polymer content, suggesting that the blend membrane becomes increasingly dense as the polymer content rises.

**Figure 7:**
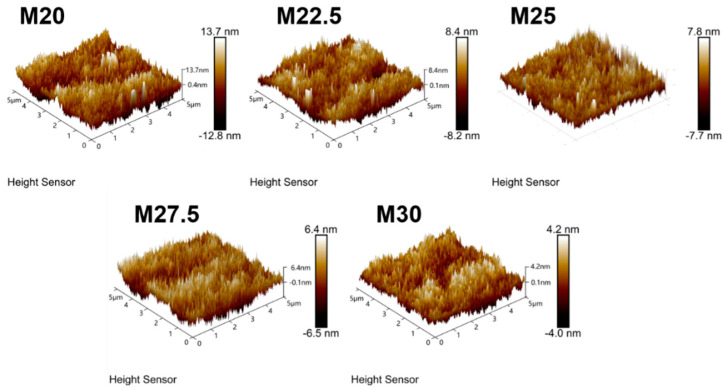
Surface 3D AFM picture of PES/SPES blend membrane.

**Figure 8:**
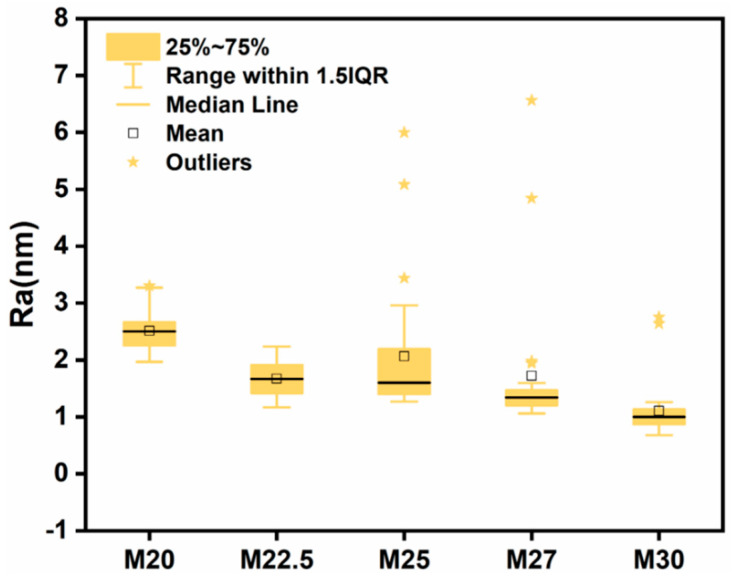
Roughness (Ra) of PES/SPES blend membrane.

AFM was employed to analyze the roughness of PES/SPES blend membranes with varying solid contents, revealing the influence of solid content on membrane roughness. Figure 7 displays the three-dimensional AFM images of PES/SPES blend membranes at different solid content levels. Figure 8 indicates that roughness decreases with an increase in solid content, with the PES/SPES blend membrane achieving its lowest roughness (Ra) of 4.2 nm at a solid content of 30%. Consistent with previous findings [22], an increase in solid content leads to a higher viscosity of the casting solution, which in turn corresponds to a reduction in the roughness of the composite membrane.

**Figure 9:**
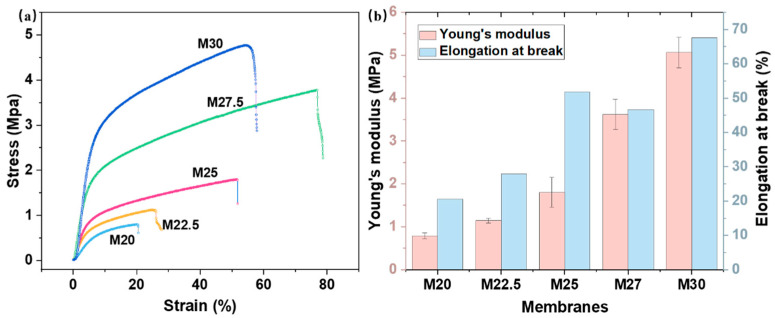
Tensile strength (**a**) and elongation at break (**b**) of PES/SPES blend membrane.

Figure 9 depicts the tensile strength and elongation at the break of PES/SPES blend membranes with different solid contents. With the increase in polymer content, the tensile strength of the blend membrane escalates from 0.79 MPa to 5.06 MPa, and the elongation at break increases from 20.55% to 67.30%. This demonstrates that the mechanical properties of the blend membrane improve as the solid content rises. The main reason for this enhancement is the increased viscosity of the casting solution due to the higher solid content, which slows down the phase separation rate and encourages the formation of sponge-like pores and the thickening of the dense layer [15]. These sponge-like dense pores confer the blend membrane with enhanced mechanical performance.

### 2.6. Pore Size Test and Calculation of PES/SPES Blend Membrane

**Figure 10:**
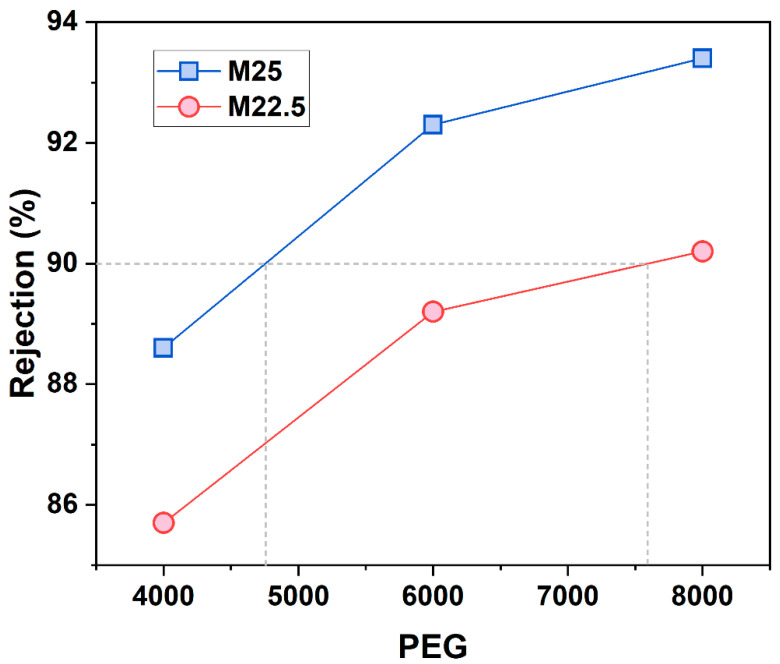
Retention rates of M22.5 and M25 blend membranes for PEG with different relative molecular weights.

MWCO serves as a metric for assessing the filtration capabilities of membranes [23]. By conducting PEG retention experiments and total organic carbon (TOC) analyses, the retention rates of the blend membranes M22.5 and M25 for PEGs of varying molecular weights can be calculated, thereby ascertaining their respective MWCOs. Figure 10 illustrates the retention performance of these blend membranes in aqueous solutions of PEG with molecular weights of 4000, 6000, and 8000. The MWCO of the blend membrane M25 is determined to be 4750, while for M22.5, it is 7600. These results substantiate the observation that an increase in solid content leads to a reduction in the pore size on the membrane surface.

### 2.7. Separation Performance of Blend Membrane

**Figure 11:**
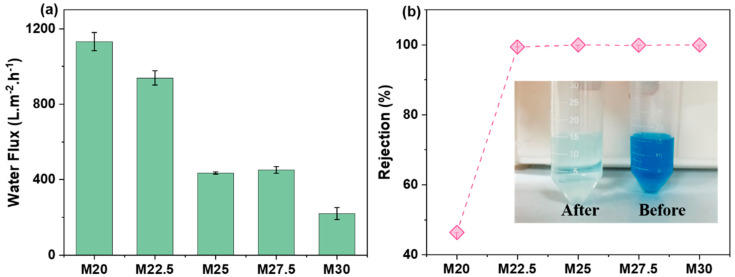
Permeation flux (**a**) and dye retention (**b**) of PES/SPES blend membrane.

Figure 11 illustrates the pure water flux and methylene blue rejection rate of the PES/SPES blend membranes. It can be observed that as the total solid content of PES and SPES in the casting solution increases, the pure water flux of the blend membrane gradually decreases from 1132 L/m^2^/h to 219 L/m^2^/h, while the rejection rate of methylene blue first increases from 50% to 99% and then steadily rises to nearly 100%. The pure water flux and dye rejection rate are closely related to the pore size and porosity of the membrane and the connectivity of the internal pores [20,24,25]. When the solid content is 20%, the casting solution is highly hydrophilic, leading to accelerated phase separation and the formation of finger-like pore structures in the blend membrane, resulting in high water flux and a low dye rejection rate. However, at higher solid contents, the viscosity of the casting solution is greater, leading to slower phase separation and the production of blend membranes with a denser skin layer and a sponge-like support layer, which reduces the membrane flux and increases the dye rejection rate.

**Figure 12:**
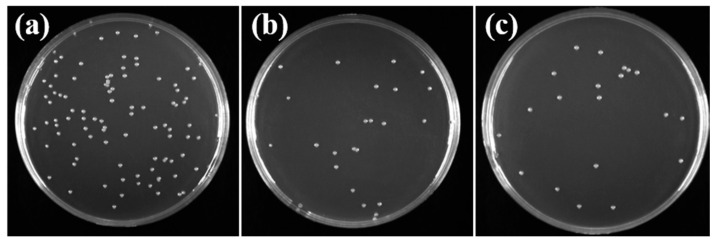
Antibacterial effects of pure PES membrane (**a**), M20 membrane (**b**) and M30 membrane (**c**).

**Figure 13:**
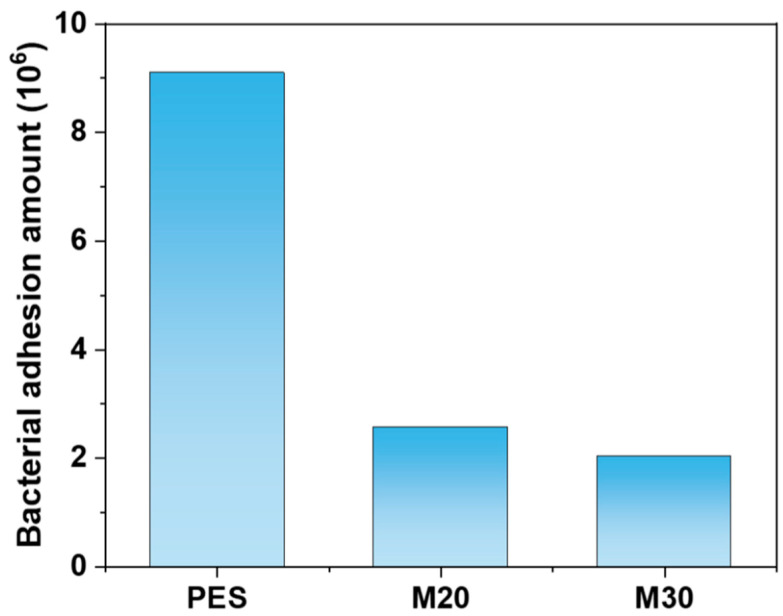
Adhesive capacities of *Escherichia coli* by pure PES membrane, M20 membrane and M30 membrane.

Figure 12 demonstrates the antibacterial efficacy of the pure PES membrane (a), M20 membrane (b), and M30 membrane (c) against microorganisms, with a focus on Escherichia coli. The pure PES membrane, after microbial adsorption, resulted in a Petri dish densely populated with microorganisms, suggesting minimal antibacterial activity. In contrast, the M20 and M30 membranes, despite having microorganisms present post adsorption, showed a substantial decrease in quantity, leading to a sparse distribution of colonies on the corresponding Petri dishes. Employing Formula (7), the adhered amounts (CFU/disk) for the three samples are depicted in Figure 13, with counts of 9.1 × 10^6^, 2.57 × 10^6^, and 2.04 × 10^6^, respectively. The reduced adsorption by SPES compared to PES is attributed to the presence of numerous sulfonic acid groups on the SPES composite membrane surface, which effectively suppress the growth of microorganisms on the membrane [26]. With an increase in solid content, the resistance of the membrane to microbial adhesion is augmented, resulting in a more pronounced inhibition of microbial growth.

## 3. Experiment

### 3.1. Experiment Materials

Reagents: PES and SPES from SINKEN (Shanghai) New Material Technology Co., Ltd. (Shanghai, China); *N*,*N*-dimethylacetamide (DMAC), polyethylene glycol (PEG), and methylene blue (MB) from Shanghai Aladdin Bio-Chem Technology Co., Ltd. (Shanghai, China); polyvinylpyrrolidone (PVP), known as Rhawn Reagent, from Shanghai Rhawn Chemical Technology Co., Ltd. (Shanghai, China). All of the above materials are AR graded.

### 3.2. Preparation of Composite Membrane

**Figure 14:**
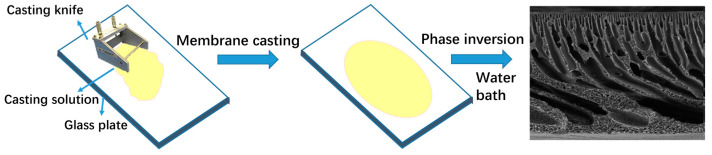
Route map of preparation of PES/SPES composite membrane.

(1) Preparation of the casting solution: firstly, following the proportions detailed in Table 2, we accurately measured the specified quantities of polymers PES and SPES, the organic solvent DMAc, and the additive PVP, and we introduced them into a pre-dried, small glass vial; subsequently, we placed the vial with the mixed solution onto a magnetic stirrer, set the stirring speed to 800 revolutions per minute (rpm), and stirred continuously for 12 h to ensure the polymers were fully dissolved; and once the dissolution was complete, we switched off the stirrer and allow the well-mixed casting solution to rest for an additional 12 h to naturally degas any residual bubbles, resulting in a homogeneous casting solution. The composite membrane was designated based on its solid content; for instance, a solid content of 20% was denoted as M20. The solid content was determined by the proportion of the total polymer (including PES, SPES, and PVP) relative to the entire casting solution. The acid% was defined as the mass ratio of the specialty chemical (SPES) to the total mass of the casting solution. For instance, if the mass of SPES was 1.28 g and the total mass of the solution was 20 g, this corresponded to an acid content of 6.4%.

(2) Preparation of composite membranes via NIPS: Drawing from methods outlined in prior studies [27,28], after the casting solution was allowed to degas and the bubbles were eliminated, it was evenly poured onto a glass plate positioned on a casting platform. A scraper with a thickness of 150 μm was utilized to spread the solution, ensuring the uniformity and precise control of the thickness of the membrane. Following the completion of the casting process, the glass plate, now coated with the casting solution, was swiftly immersed vertically into a pre-prepared pure water coagulation bath, with the water level ensuring full coverage of the height of the glass plate. This facilitated the biphasic diffusion process between the water and solvent, leading to the solidification and shaping of the membrane. Once the membrane hardened on the glass plate and spontaneously detached, it was transferred to deionized water for a 24 h soak to eliminate any residual additives and solvents. The preparation process is depicted in Figure 14.

### 3.3. Physical and Chemical Performance Characterization of Composite Membranes

#### 3.3.1. Fourier Transform Infrared Spectroscopy (FTIR) and X-Ray Photoelectron Spectroscopy (XPS) Test

In this research, Nicolet iS10 FTIR from Thermo Fisher Scientific (Waltham, MA, USA) was used to perform a comprehensive spectral analysis on the surface of the composite membranes. The spectral scanning was conducted over a range of 500 cm⁻^1^ to 4000 cm⁻^1^. Additionally, the Thermo Scientific K-Alpha X-ray Photoelectron Spectrometer (XPS, Thermo Scientific K Alpha, Thermo Fisher Scientific Waltham, MA, USA, Energy range: 0–5000 ev) was used for the semi-quantitative analysis of the elemental composition and content within the composite membranes. From the XPS spectra, information about the elemental composition, chemical states, and molecular structures on the sample surface was derived, with peak intensities providing insights into the surface elemental content.

#### 3.3.2. Viscosity Test

The instrument used in this experiment was DV2-T (from Brookfield, WI, USA), and the samples were tested at a temperature of 25 °C and a revolution speed of 60 RPM.

#### 3.3.3. Porosity Test

In this research, the dry–wet weight method is utilized for the quantitative analysis of the porosity of the membrane. The procedure is conducted as follows: initially, trim the wet membrane sample to possess a defined surface area; then, meticulously remove any moisture from both the top and bottom surfaces of the membrane using lens paper, and precisely record the mass of the wet membrane; subsequently, place the wet membrane in an oven preheated to 60 °C and dry it for 12 h to ensure the complete evaporation of the moisture within the membrane; after drying, re-weigh the membrane to obtain its dry mass; and lastly, with Formula (1), input the wet membrane mass, dry membrane mass, and the known membrane density to compute the porosity of the membrane. The formula is as follows:

(2) $$\delta =\frac{W_{w}-W_{d}}{Sd\rho_{w}}\times 100\%$$

where δ is porosity; *W_w_* and *W_d_* denote the wet weight and dry weight of the membrane, g; *d* is the average thickness of the tested sample membrane, cm; *S* is the surface areas of the tested sample membrane, cm^2^; and $\rho_{w}$ is the density of water, g/cm^3^.

#### 3.3.4. Contact Angle Test Characterization

The steps were to select samples with dimensions no smaller than 2 mm × 2 mm and position them on the testing platform of the JY-82 model contact angle meter; we employed the automatic dispensing feature of the instrument to meticulously apply an exact quantity of a reagent, such as a water droplet, onto the surface of the sample; and then we captured a photograph of the surface of the sample and used the goniometric method to precisely measure and document the contact angle value.

#### 3.3.5. SEM Characterization

The steps were as followed: When examining the surface of the membrane, we cut samples of a specific size and free from defects and then securely fixed them onto a sample stage with conductive adhesive. For the examination of the cross-section of the membrane, the membrane was first fractured into appropriately sized samples using liquid nitrogen, followed by attachment to the sample stage. Furthermore, all membranes should be subjected to gold sputtering treatment before testing to improve their electrical conductivity. This experiment utilized a scanning electron microscope, the Regulus 8100 model from Hitachi Corporation in Tokyo, Japan, to perform microscopic observations of both the surfaces and cross-sections of the prepared composite nanofiltration membranes.

#### 3.3.6. Atomic Force Microscopy (AFM) Characterization

AFM determined the height variations on the membrane surface by directly measuring the force applied to the probe. It evaluated the surface roughness and micromorphology through parameters such as the arithmetic mean roughness and the root mean square roughness. For this experiment, a Bruker Dimension ICON atomic force microscope from German Bruker Corporation (Bremen, Germany) was employed to characterize the micromorphology and roughness of the membrane.

#### 3.3.7. Mechanical Performance Characterization

In compliance with the GB13022-1991 standard [29], the mechanical strength of the membrane was evaluated using a tensile testing machine (CMT6103 from MTS Industrial Systems, Eden Prairie, MN, USA). Before testing, the membranes were conditioned by drying at room temperature for 24 h. The self-supporting flat samples were cut into a dumbbell shape, and the test was conducted with a tensile speed of 20 mm/min.

#### 3.3.8. Molecular Weight Cut-Off (MWCO) Test

The MWCO and average pore diameter of the blend membranes M22.5 and M25 were determined using PEG solutions with molecular weights of 4000, 6000, and 8000. Retention tests were performed on these PEG solutions at a concentration of 0.1 g/L under a pressure of 1 bar. The MWCO of the membrane was defined as the molecular weight of PEG at which 90% retention was achieved.

### 3.4. Separation Performance Test of Composite Membranes

#### 3.4.1. Permeability Test

**Figure 15:**
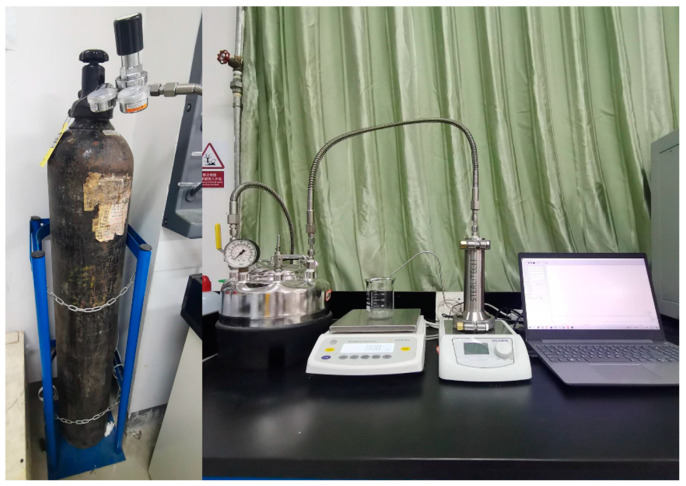
Diagram of membrane performance test apparatus [30].

The testing procedure, as referenced from prior research [30], is detailed here: The test membrane was installed in the terminal filtration setup depicted in Figure 2. Deionized water served as the feedstock, and the permeability of the composite membrane was assessed by determining the membrane flux. For flux testing, the experiment was repeated with five samples of each membrane under identical conditions, and the average value was computed. The experiment utilized a dead-end filtration apparatus, as illustrated in Figure 15, which comprised a nitrogen cylinder, a sealable water basin, a filtration chamber, and a digital scale. The filtration chamber has a volume of 300 mL with an effective diameter of 0.032 m.

The filtration procedure is outlined below: (1) the membrane was first subjected to a transmembrane pressure of 2 bar for 2–3 h to attain a consistent flux; (2) after the flux was stabilized, the transmembrane pressure was adjusted to 1 bar, and the pure water flux was recorded every 30 s, accumulating at least 50 measurements, from which the average flux value was derived. The calculation of the membrane flux was based on the following formula:

(3) $$J=\frac{V}{Τ\times A}(L/m^{2}\cdot h)$$

where *J* is the membrane flux, L/m^2^·h; *V* is the filtration volume, *L*; T is the filtration time, h; and A is the effective area of the membrane, m^2^. Specifically,

(4) $$V=\frac{m}{\rho }=\frac{\Delta xg}{1000g/L}=\frac{\Delta x}{1000}(L)$$

(5) $$Τ=\frac{\Delta t}{3600}=\frac{30}{3600}=\frac{1}{120}(h)$$

(6) $$A=\frac{\pi }{4}d^{2}=\frac{\pi }{4}\times {\left(0.032\right)}^{2}(m^{2})$$

Thus, the flux formula is as follows:

(7) $$J=149.28\Delta x(L/m^{2}\cdot h)$$

#### 3.4.2. Dye Retention Rate

A single dye (MB) solution with a concentration of 10 mg/L was used as the feed to assess the retention performance of the membrane. The retention test employed the same setup as the aforementioned permeability test. The retention rate testing procedure is detailed below: (1) Select several membranes from the flux tests that are close to the average value for retention rate testing. (2) Ensure the sealed water tank is empty, and add 200 mL of the solution into the 300 mL filtration chamber; place a rotor inside to maintain a revolution speed of 600 rpm under the influence of a magnetic stirrer to mitigate concentration polarization on the membrane surface. (3) Set the transmembrane pressure to 1 bar, and filter for 1 h; discard the first 20 mL of filtrate and collect 30 mL of filtrate for the calculation of the dye retention rate; repeat the test under identical conditions for the original solution and filtrate of three parallel membranes; and calculate the average value to minimize experimental error. The filtrate was added into the cuvette of the test platform; then, the absorbance of the filtrate was measured by using the quantitative measurement function of the spectrophotometer (T9S, Beijing Puyang General Instrument Co., LTD, Beijing, China) after calibration.

The retention rate was calculated using the following formula:

(8) $$R=\frac{C_{0}-C_{1}}{C_{0}}$$

where *R* is the retention rate; *C*_0_ is the concentration of raw material solution (mg/L); and *C*_1_ is the concentration of filtered fluid (mg/L).

#### 3.4.3. Antibacterial Experiment

This experiment utilized *Escherichia coli* as the target bacterial strain to assess the antibacterial capabilities of pure PES membranes (control group), M20 membranes, and M30 membranes, comprising a total of three sample groups. The specific operational steps for the test are outlined below: Initially, 10 mL of a bacterial suspension diluted to a concentration of 10^6^ CFU/mL with sterile PBS was added to the sample tubes and incubated at 37 °C in a shaking incubator for 6 h. Post incubation, the samples were retrieved and rinsed three times with sterile PBS to eliminate microorganisms not adhering to the membrane surface. Subsequently, the samples were submerged in 10 mL of sterile PBS and sonicated for 7 min to detach the microorganisms adsorbed onto the sample surfaces into the sterile PBS solution. Thereafter, 100 μL of the eluate, which had been diluted tenfold, was evenly spread onto LB agar plates and incubated at 37 °C for 18 h. Finally, the plates were photographed, and the colony count was documented following the principles outlined in GB4789.2-2016 [31]. The antibacterial performance was evaluated based on the quantity of bacterial adhesion (CFU/disk), with the formula provided as follows:

(9) $$CFU=N\cdot D\cdot V_{a}\cdot V_{b}$$

where *N* is the *CFU*; the dilution ratio is *D*; *V*_a_ is the total coating volume, which is 10 mL in the experiment; and *V*_b_ is the total volume of the eluted bacterial solution of sterile PBS solution, which is 10 mL in the experiment.

## 4. Conclusions

PES/SPES blend membranes were prepared using the non-solvent-induced phase inversion method, and the study examined the effects of solid content on the micromorphology and separation performance of these membranes. Compared to pure PES membranes, the PES/SPES blend membranes demonstrated an enrichment of the hydrophilic sulfonic acid groups on their surfaces and exhibited greater density. As the solid content increased, the porosity of the blend membranes decreased from 10.8% to 4.06%, while the tensile strength rose from 0.79 MPa to 5.06 MPa. Additionally, the rejection rate of methylene blue improved from 50% to nearly 100%. The findings indicated that the addition of SPES enhanced the resistance of blend membranes to Escherichia coli adhesion, effectively inhibiting microbial growth. Furthermore, a higher content of sulfonic acid groups correlated with improved antibacterial properties of the composite membrane.

## Figures and Tables

**Table 1:** Polymer parameters [20].

Polymer	Density ρ/(g·cm^2^)	Unit Molar Mass M/(g·mol)	Solubility Parameter δ/(J^1/2^/mol^1/2^)
PES	1.37	232	11.0
SPES	1.25	256	11.6

**Table 2:** Formula design of casting solution.

Number	Polymer Content %	PES/g	SPES/g	PVP/g	DMAc/g
M20	20	1.92	1.28	0.8	16
M22.5	22.5	2.22	1.48	0.8	15.5
M25	25	2.52	1.68	0.8	15
M27.5	27.5	2.82	1.88	0.8	14.5
M30	30	3.12	2.08	0.8	14

## Data Availability

The data presented in this study are available on request from the corresponding author.
